# Maternal Condition Does Not Influence Birth Sex Ratios in Anubis Baboons (*Papio anubis*)

**DOI:** 10.1371/journal.pone.0012750

**Published:** 2010-09-22

**Authors:** Joan B. Silk, Shirley C. Strum

**Affiliations:** 1 Department of Anthropology and Center for Society and Genetics, University of California Los Angeles, Los Angeles, California, United States of America; 2 Department of Anthropology, University of California San Diego, San Diego, California, United States of America; 3 Uaso Ngiro Baboon Project, Nairobi, Kenya; Texas A&M University, United States of America

## Abstract

Trivers and Willard predicted that when parental condition has differential effects on the fitness of male and female offspring, parents who are in good condition will bias investment toward the sex that benefits most from additional investment. Efforts to test predictions derived from Trivers and Willard's model have had mixed results, perhaps because most studies have relied on proxy measures of parental condition, such as dominance rank. Here, we examine the effects of female baboons condition on birth sex ratios and post-natal investment, based on visual assessments of maternal body condition. We find that local environmental conditions have significant effects on female condition, but maternal condition at conception has no consistent relationship with birth sex ratios. Mothers who are in poorer condition at the time of conception resume cycling significantly later than females who are in better condition, but the sex of their infants has no effect on the time to resumption of cycling. Thus, our findings provide strong evidence that maternal condition influences females' ability to reproduce, but females do not facultatively adjust the sex ratio of their offspring in relation to their dominance rank or current condition.

## Introduction

When parental condition has differential effects on the fitness of male and female offspring at weaning, and variation in condition at weaning persists into adulthood, parents who are in good condition are expected to bias investment toward the sex that benefits more from additional investment [1]. In most sexually dimorphic species, large body size enhances male reproductive success, and males are likely to benefit more from additional investment than females [2]. Therefore, in these species, parents in good condition are expected to bias investment in favor of sons. Efforts to test Trivers & Willard's model have produced a mixed pattern of results. In a meta-analysis of more than 400 studies on mammalian species, Cameron [3] found that about one-third of the studies provided significant support for the Trivers and Willard model. Most of the remaining studies showed no consistent relationship between maternal condition and offspring sex, and a few found significant patterns that were in the opposite direction.

Nearly all of these studies relied on proxy measures such as maternal dominance rank, parity, litter size, and food availability, that were assumed to be related to physical condition. Cameron found that the small subset of studies that relied on measures of maternal condition, such as body weight, at the time of conception produced substantially stronger support for the Trivers and Willard model: in 22 of these 25 studies, females in good condition produced substantially more sons than females in poor condition. Based on these findings, Cameron suggested that “sex-adjustment occurs at or near implantation” and concluded that the inconsistency of results in the literature may be largely due to the use of inaccurate proxies for maternal condition.

Cameron's meta-analysis raises interesting questions about facultative adjustments of birth sex ratios in nonhuman primate groups. There have been more than three dozen studies of facultative birth sex ratios in a range of nonhuman primate species, and nearly all have relied on maternal dominance rank as an indirect measure of maternal condition. Some studies showed that high-ranking females produce significantly more sons than daughters, while others showed the opposite pattern, and some showed no consistent pattern at all. Two meta-analyses of somewhat different sets of these studies demonstrated that the difference in the birth sex ratios of high- and low-ranking females declined as the sample size increased [4], [5], suggesting that the variation represented stochastic effects of small samples. Schino [5] reports that the difference in the proportion of males produced by high- and low ranking females is significantly positively related to population growth rate. However, the significance of this relationship relies on a single data point based on one group of baboons [5]. A subsequent analysis of data from 36 groups of baboons which incorporated the effects of local resource competition on birth sex ratios failed to demonstrate consistent evidence for facultative adjustment of birth sex ratios [6].

Cameron's results suggest that more consistent results might be obtained with more accurate measures of maternal condition. To date, only two studies of cercopithecine primate species have examined the effects of maternal condition rather than dominance rank to test the predictions of the Trivers and Willard model. In free ranging toque macaques (*Macaca sinica*) in Sri Lanka, high ranking females were generally heavier than lower ranking females [7]. However, females in good and poor condition both produced significantly more sons than daughters, while females in moderate condition produced significantly more daughters than sons. Among rhesus macaques (*Macaca mulatta*) on Cayo Santiago Island, middle-ranking females produced significantly more daughters than higher- or lower-ranking females did [8]. However, female dominance rank was unrelated to female adiposity, and there was no difference in the adiposity of females who produced sons and daughters. It is possible that provisioning in these animals reduces the effects of variation in competitive ability, and obscures the relationship between dominance rank, female condition, and offspring sex. Thus, present evidence suggests that maternal condition has no consistent impact on birth sex ratios, but leaves open the possibility that maternal condition might influence the extent of investment in male and female offspring.

Here, we present the results of an analysis of the relationship between maternal dominance rank, maternal condition, and birth sex ratios in a wild population of baboons, *Papio anubis.* Previous work indicated that maternal dominance rank was not consistently related to birth sex ratios in this population [4], [9]. Here, we extend these analyses in several ways. First, we show that female condition at conception varies in relation to ecological conditions. Second, we test the prediction that females in good physical condition will produce more sons than females in poor condition. Third, we test the prediction that females in good condition will invest more in sons than daughters, using the duration of the period between birth and the resumption of cycling as a proxy for the extent of investment.

## Methods

The study used data from females in groups of wild baboons living on the eastern Laikipia Plateau of central Kenya. Group histories are dynamic. Two study groups (PHG, MLK) were translocated from the Rift Valley near Gilgil, Kenya (Kekopey) to the Laikipia region in 1984 [10]. The third study group (STT), added in 1985, was indigenous to the translocation site [10], [11]. One translocated group, MLK, fused with the indigenous troop, STT, in 2001 to form the fourth study group (NBO). Although PHG had been monitored since 1971, MLK since it fissioned from PHG in 1981, and STT since 1985, the data in this study are limited to the period for which information on female body condition was available, 1993 through 2008.

The study groups range in an area that is topographically diverse and averages 1718 m above sea level [12]. Rainfall is typically concentrated during two wet seasons (March-June, November-December; Barton 1992, 1993). Annual rainfall calculated from 1985 to 2003 averaged 350 mm [10]. The baboons' habitat is dry savanna which includes grassy plains, acacia woodlands and dry forests situated along sand rivers. Recently, *Opuntia stricta*, a non-indigenous cactus, has invaded the area.

Baboons feed on a variety of grasses, herbs, sedges, and the flowers and fruits/pods of a variety of shrubs and trees including several *Acacia* species. Succulents make up a significant part of the diet (Strum, unpublished data); these include *Sansevieria*, *Euphorbia*, and *Opuntia* species. Seasonality is an important feature. Food availability generally declines in the dry season [13]. Droughts are frequent.

### Demographic Information

The data comprise information about births, deaths, and female reproductive status extracted from the project records. Information about maternal age at conception was not available for the original indigenous STT females. Birth and death dates of mothers and their infants were used to compute maternal age at the time each infant was born and to determine how long each infant lived. For full term births, the date of conception was computed by subtracting 177 days from the date of birth [14], [15]. The analyses are limited to females that gave birth to full-term infants because the sex of infants that were aborted or miscarried during a pregnancy is unknown.

We calculated the number of days that elapsed between the birth of an infant and the resumption of a female's estrous cycling using project records on female reproductive condition. Time to resumption of cycling may be over-estimated if cycles are missed or subsequent pregnancies are not detected. To exclude this possibility, we identified outliers using box plots; intervals following birth of surviving and non-surviving infants were considered separately. For infants that died within six months, values that exceeded 280 days were excluded (n = 2). For infants that survived six months, no outliers were detected.

Information about time to resumption of cycling was unavailable for 7 infants who died before they were 100 days of age. Their mothers would have been expected to resume cycling within about 94 days (see results). Only two of these mothers survived more than 100 days after they delivered their last infants. Information about time to resumption of cycling was unavailable for 16 infants who survived at least 100 days. Their mothers would have been expected to resume cycling about 293 days on average (see results). Only 6 of these mothers lived more than 300 days after they delivered their last infants. Taken together, the number of intervals that were censored due to maternal deaths was 8.

### Assessment of Maternal Rank

Dominance ranks were assessed on a monthly basis using daily ad libitum observations of avoidance and aggression between females scored on a one/zero basis per day per category of behavior. Females were arranged into a linear dominance hierarchy by minimizing the number of reversals below the diagonal. Females are categorized as high-ranking (top third of the dominance hierarchy), middle-ranking (middle third), or low-ranking (lower third). Dominance relationships among females have remained relatively stable across time.

### Assessment of Maternal Condition

Visual assessments of maternal condition were made four times per month using calibrated photographs to score different body parts. Observers recorded the condition of (1) ischial callosities (extent of protrusion and flaking), (2) visibility of the ribs, (3) visibility of the pelvis, (4) tail stiffness, and (5) extent and location of hair loss. Each feature was scored independently from 0 (best) to 5 (worst).

Baboons' pelvis and ribs are not seen normally but both become more visible when animals experience hair loss and weight loss associated with decline in condition. In addition, water and fat are lost from the paracallosal area making the ischial callosities protrude. The callosities themselves may begin to flake as the hair that normally forms the hardened pads begins to disintegrate. Finally, baboons' tails may become stiff and inflexible.

We extracted the mother's condition records during the week of conception. If no condition records were available for the week of conception, then we used the condition records for the week prior to conception. If information about the week before conception was also missing, then we checked for records two weeks before conception and one week after conception. If these two values were the same, we used these values; if the values differed, we treated this as missing information. Information was available for 78% of infants conceived during the study period.

The scores for the five condition features were highly inter-correlated, and a factor analysis confirmed that all of the features loaded onto a single factor. Therefore, we computed the average value of the individual scores to obtain an aggregate measure of female condition. Low values correspond to females in good condition, and high values correspond to females in poor condition.

### Assessment of Biomass

Herbaceous biomass was measured monthly using the slanting pin intercept technique angled 65° from vertical [16] and converted into biomass in gr/m^2^ using the adjusted equation HB = total hits ×0.847 [16], [17]. Values of biomass during the month of conception and the month before conception were highly correlated (r = 0.9411, p<0.0001, n = 199). We used biomass during the month of conception in the analyses reported below.

### Data Analysis

The dataset includes information about 79 females who gave birth to 204 infants. Because some females gave birth to multiple infants, the data are not independent. Thus, we used Generalized Linear Mixed Models with mother's identity as a random factor. When the dependent variable was continuous (mother's condition, time to resumption of cycling) we used a linear regression form of the GLMM, and when the dependent variable was binary (infant sex) we used the logistic regression form of the GLMM.

The GLMM assumes that residuals are normally distributed and homogenous. For each model that we constructed, we examined the distribution and homogeneity of residuals. Residuals were plotted against the fitted values to determine whether the distribution of the fitted values were similar along the entire range of residual values. We detected no strong evidence of deviations from normality or homogeneity.

Preliminary analyses showed that biomass at conception and maternal age had nonlinear effects on maternal condition. To reduce colinearity between the linear and quadratic terms, these variables were centered on the mean. We evaluated potential interactions among predictor variables (e.g. condition x maternal rank), but found no significant effects, and dropped interaction terms from the models reported here.

All analyses were conducted with STATA 11.0 (StataCorp 2009). All statistical tests are two-tailed. Means and standard deviations are given, where appropriate. Sample sizes vary across analyses because information about maternal age was not available for all females.

## Results

### Maternal Condition at Conception

Information about condition at conception was available for 79 females, ranging in age from 5.4 to 22.3 years, who produced 204 full-term infants. Condition values for conceiving females ranged from 0 to 3, with a mean and standard deviation of 1.23±0.60.

**Figure 1:**
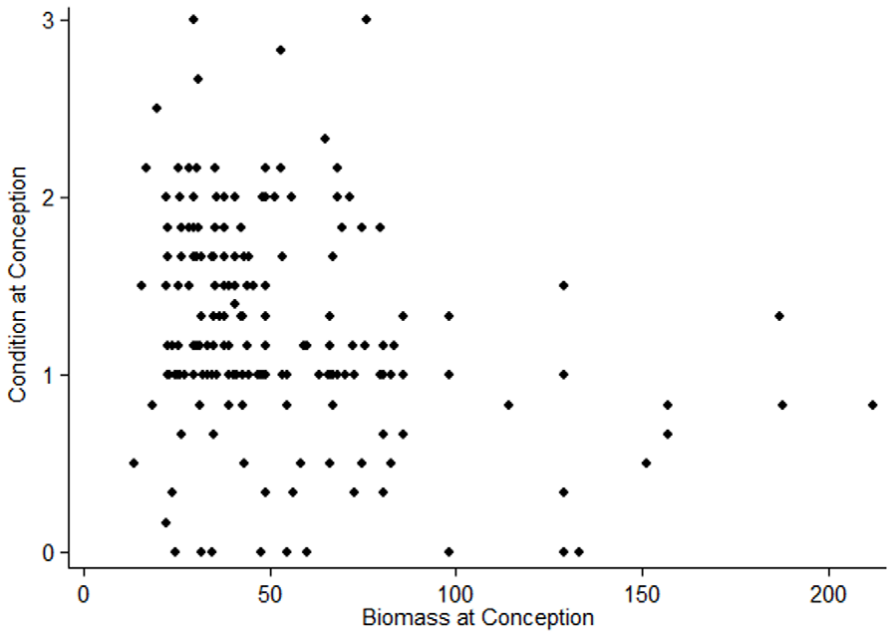
Biomass influences maternal condition at conception. Maternal condition is scaled from 0 (best) to (5) worst, so low values represent females in relatively good condition.

**Figure 2:**
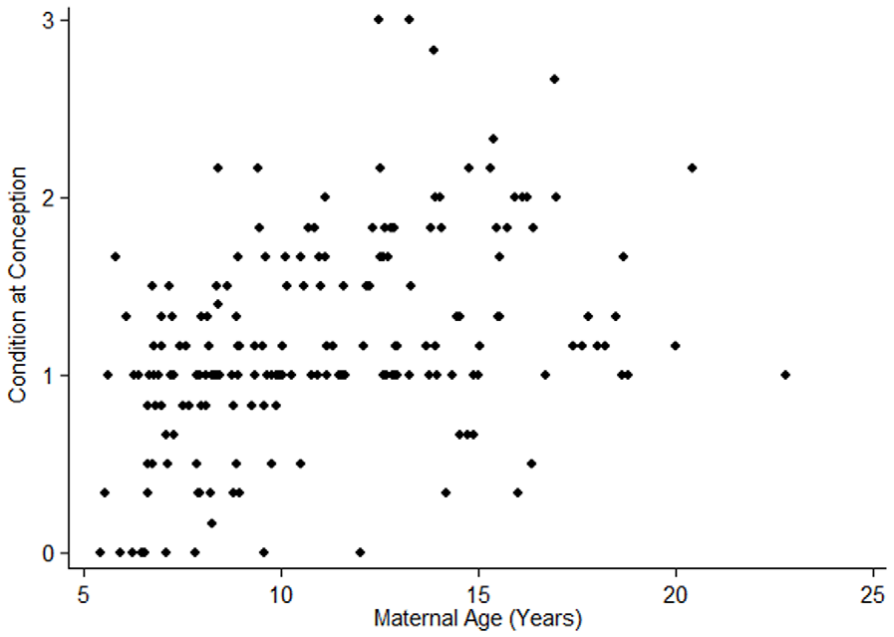
Maternal age is associated with maternal condition at conception. Maternal condition is scaled from 0 (best) to (5) worst, so low values represent females in relatively good condition.

Females' condition tracked environmental conditions and age, but not their dominance rank (Wald χ^2^ = 76.17, p = <0.0001, n = 176; Table 1). Females were in significantly better condition at conception when biomass values were higher (Figure 1). Females' condition at conception generally declined as they got older, but this effect was attenuated among females older than about 15 years of age (Figure 2). There was no consistent effect of female dominance rank on condition at conception.

**Table 1:** Sources of variation in condition at conception.

Predictor Variables	Coefficient	Standard Error	z	p
Biomass	−0.0073	0.0016	−4.59	<0.001
Biomass^2^	0.00006	0.00002	3.20	0.001
Maternal age	0.0844	0.0112	7.52	<0.001
Maternal age^2^	−0.0089	0.0023	−3.86	<0.001
Dominance rank	−0.0329	0.0433	−0.76	0.447

### Birth Sex Ratio

Females whose condition was known at the time of conception gave birth to 94 females and 108 males, a distribution that did not differ significantly from unity (Binomial probability test, p = 0.3604; 2 unknown sex) or from the distribution of males and females in the full data set (z = −0.23, p = 0.818). The birth sex ratios of high-ranking, middle-ranking, and low-ranking females in this sample did not differ significantly from unity (HR, 38 males: 42 females, p = 0.5250; MR: 37:26, p = 0.5873; LR: 33:26, p = 0.5593). Moreover, the proportion of males born to females in different rank categories did not differ (Two-sample test of proportions: HR vs MR, p = 0.1819; HR vs LR, p = 0.3256; MR vs LR, p = 0.7548).

There was no significant relationship between sex of infant and maternal condition or maternal dominance rank (Wald χ^2^ = 3.03, p = 0.2202, n = 202; Table 2).

**Table 2:** Factors influencing infant sex.

Predictor Variables	Coefficient	Standard Error	z	p
Condition	−0.3716	0.2618	−1.42	0.156
Rank	0.6362	0.1749	1.02	0.310

### Time to resumption of cycling

As expected, mothers resumed cycling considerably later if their infants survived the first six months of life than if their infants died during this period (surviving infants: 293.35±118.4 days, n = 140, no outliers; non-surviving infants: 94.42±56.58, n = 36).

**Figure 3:**
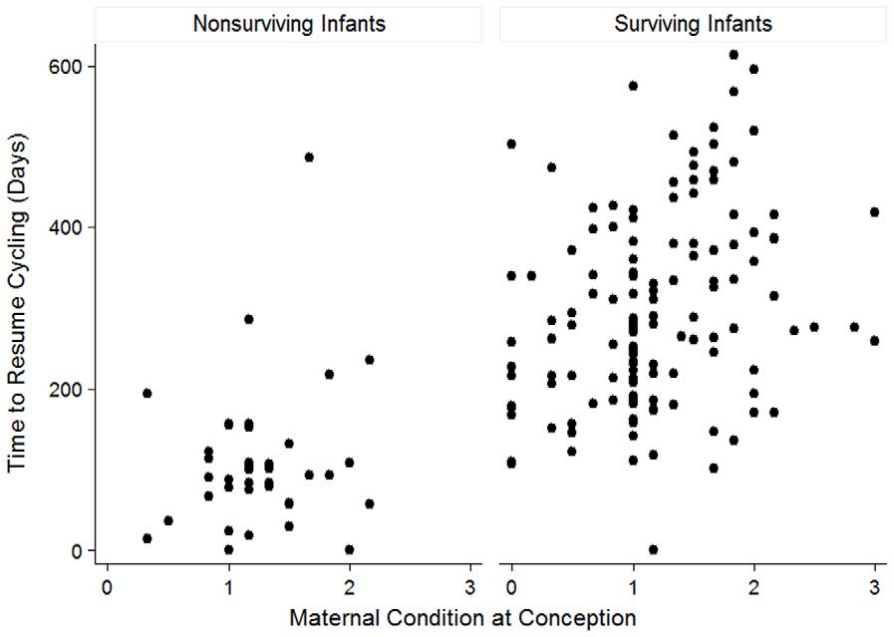
Maternal condition at conception influences time to resumption of cycling after the birth of (a) nonsurviving and (b) surviving infants.

**Figure 4:**
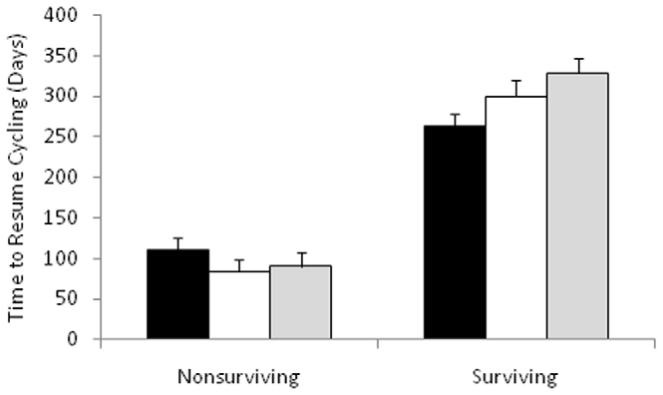
Female dominance rank influences time to resumption of cycling after birth of (a) surviving and (b) nonsurviving infants. High rank  =  black bars, middle rank  =  grey bars, low rank  =  white bars.

We considered the effects of infant sex, maternal condition at conception, and maternal rank on the time to resumption of cycling (Wald χ^2^ = 127.58, p = <0.0001, n = 176). Infant sex did not have a consistent impact on the time to resumption of cycling (Table 3). However, mothers who were in better condition at the time of conception resumed cycling significantly sooner than females who were in worse condition, when the effects of other variables were controlled (Table 3; Figure 3). In addition, high ranking females resumed cycling significantly earlier than lower ranking females, and this effect was particularly pronounced after the birth of surviving infants (Figure 4).

**Table 3:** Sources of variation in time to resumption of cycling.

Predictor Variables	Coefficient	Standard Error	Z	p
Infant survival	20.0173	19.5160	10.25	<0.001
Condition	43.6068	14.1891	3.07	0.002
Infant sex	−10.5519	15.4353	−0.68	0.494
Rank	26.5687	9.4895	2.80	0.005

## Discussion

There have now been three studies of facultative adjustment of birth sex ratios in cercopithecine primate species that were based on measures of female condition rather than dominance rank [7,8, this study]. None of these studies have found that mothers of males are in significantly better condition at conception than mothers of females. It is possible that with larger samples that generate greater statistical power, or more accurate measures of maternal condition, more consistent patterns would emerge. However, current evidence indicates that cercopithecine primates represent an exception to Cameron's [3] finding that analyses which are based on direct measures of maternal condition consistently support the Trivers and Willard model.

It seems possible that facultative adjustment of birth sex ratios has evolved in some mammalian taxa, but not others. Nearly half of the samples that showed a significant relationship between maternal condition at conception and offspring sex in Cameron's metaanalysis were derived from studies of rodents [from Appendix A]. Six additional studies were conducted on ungulates, four on marsupials, two on pinnipeds, and two on primates. The studies of rodents and ungulates consistently supported the prediction that females in good condition will produce more sons than females in poor condition, but the patterns were less consistent for marsupials, pinnipeds, and primates.

It is possible that variation in the relationship between maternal condition and offspring sex across taxa is linked to differences in life history strategies. Condition at conception may have a more consistent impact on investment capacity in capital breeders, which use stored energy to sustain their offspring, than income breeders, which use energy that they obtain during gestation and lactation to nourish their offspring [18]. For income breeders, condition at conception would only reflect females' investment capacities if their condition at conception was consistently correlated with their ability to secure resources during important stages of offspring development. The capital-income breeding dichotomy seems to fit evidence for ungulates. The four ungulate taxa included in Cameron's analysis (reindeer, red deer, elk, and fallow deer) are all considered to be capital breeders [19]. Another ungulate, the European roe deer is an income breeder, and shows much less consistent relationships between maternal condition and offspring sex [20]. However, the rodents in Cameron's sample showed consistent relationships between maternal condition and offspring sex although they are generally classified as income breeders. Thus, the dichotomy between capital breeders and income breeders may not provide a simple explanation for variation in the relationship between maternal condition and offspring sex.

Although Cameron reasoned that maternal condition provided a more accurate measure of mothers' ability to invest in offspring than maternal rank, Sheldon and West [20] came to the opposite conclusion. They conducted a meta-analysis of the relationship between maternal dominance rank, condition, and birth sex ratios in ungulates using 37 studies on 18 species. Like Cameron, they found that mothers in good condition were more likely to produce male offspring than mothers in poor condition. However, effect sizes derived from studies based on maternal rank were considerably larger than effect sizes based on direct measures of maternal condition at conception. Sheldon and West suggest that this may be because maternal dominance rank is a better predictor of female ungulates' ability to secure resources for themselves and their offspring in the future than is their current condition. The data presented here, along with previous meta-analyses which found no consistent difference between birth sex ratios of high- and low-ranking females [4]–[6], suggest that cercopithecine primates, including baboons, do not facultatively adjust the sex of their progeny. This is somewhat surprising because they seem to fit the assumptions underlying Trivers & Willard's model. For example, baboons have pronounced sexual dimorphism [21] males compete actively for access to conceiving females; male dominance rank is closely related to condition; and there is considerable skew in male lifetime reproductive success in [22]. These are the kinds of conditions in which mothers in good condition are expected to bias investment in favor of sons.

It is possible that primates lack the physiological capacity to manipulate birth sex ratios. This is difficult to evaluate because the proximate mechanisms that regulate facultative adjustment of birth sex ratios have not been identified. A number of candidates have been suggested, including maternal glucose levels [3] and timing of insemination [23]. There is no *a priori* reason that these mechanisms would be effective in rodents and ungulates, but not in primates.

It is also possible that baboon mothers have less impact on their offspring's condition at weaning and in adulthood than ungulate and rodent mothers do. In red deer, for example, mothers' condition influences sons' condition at weaning and in adulthood [24]. It is not clear whether this is true of baboons and other cercopithecine primates [7]. Baboons mate throughout the year and experience considerable fluctuation in food availability within and across years. Our data show that maternal condition at conception is significantly affected by current environmental conditions, but variation in environmental conditions across time may weaken the link between mother's condition at conception and her offspring's condition at weaning. Sons' condition at weaning may not be a strong predictor of their competitive ability when they disperse to non-natal groups and begin to reproduce at the age of about 8–9 years [22]. Male dominance rank is linked to physical condition, but there is considerable variation in the relationship between male rank and reproductive success within and across groups [22], [25]. A previous study of one of our study groups demonstrated that high ranking males did not have priority access to estrous females or meat, and emphasized the importance of the quality of male-female relationships in male mating strategies [25]. Thus, mothers in good condition may not consistently benefit from allocating extra investment to their sons.

Mothers might have more control over the condition of their daughters than their sons. Daughters acquire their mothers' dominance rank and remain in their natal groups throughout their lives. Although maternal rank did not influence females' condition at the time of conception, high ranking females that produced surviving infants resumed cycling significantly earlier than lower ranking females did. Similar patterns have been documented among the Amboseli baboons [26]. This suggests that high ranking females might forage more efficiently, have access to higher quality resources, or expend less energy in caring for their infants than lower ranking females do. However, dominance rank has relatively modest effects on the reproductive success of females in most populations [27].

The Trivers and Willard model relies on the assumption that one sex benefits more from additional investment than the other, and that offspring condition at the end of the period of maternal investment is correlated with offspring condition and competitive ability in adulthood. For animals, like baboons, that mature slowly, or breed throughout the year and live in seasonal and variable environments, the second assumption may be particularly difficult to fulfill. If this is the case, then selection may not favor facultative adjustment of birth sex ratios in relation to maternal condition. More research is needed to determine whether baboon mothers' condition influences the condition of their offspring at weaning, and whether variation in condition at weaning extends into adulthood.
